# Personalized Multimodal Lifestyle Intervention as the Best-Evidenced Treatment for Chronic Pain: State-of-the-Art Clinical Perspective

**DOI:** 10.3390/jcm13030644

**Published:** 2024-01-23

**Authors:** Jo Nijs, Anneleen Malfliet, Eva Roose, Astrid Lahousse, Wouter Van Bogaert, Elin Johansson, Nils Runge, Zosia Goossens, Céline Labie, Thomas Bilterys, Jente Van Campenhout, Andrea Polli, Arne Wyns, Jolien Hendrix, Huan-Yu Xiong, Ishtiaq Ahmed, Liesbet De Baets, Eva Huysmans

**Affiliations:** 1 Pain in Motion Research Group (PAIN), Department of Physiotherapy, Human Physiology and Anatomy, Faculty of Physical Education & Physiotherapy, Vrije Universiteit Brussel, 1090 Brussels, Belgium; anneleen.malfliet@vub.be (A.M.); eva.charlotte.s.roose@vub.be (E.R.); astrid.lucie.lahousse@vub.be (A.L.); wouter.van.bogaert@vub.be (W.V.B.); elin.johansson@vub.be (E.J.); nils.arno.andreas.runge@vub.be (N.R.); zosia.goossens@vub.be (Z.G.); celine.labie@kuleuven.be (C.L.); thomas.bilterys@vub.be (T.B.); jente.van.campenhout@vub.be (J.V.C.); andrea.polli@vub.be (A.P.); arne.wyns@vub.be (A.W.); jolien.hendrix@vub.be (J.H.); huanyu.xiong@vub.be (H.-Y.X.); ishtiaq.ahmed@vub.be (I.A.); liesbet.de.baets@vub.be (L.D.B.); eva.huysmans@vub.be (E.H.); 2 Chronic Pain Rehabilitation, Department of Physical Medicine and Physiotherapy, University Hospital Brussels, 1090 Brussels, Belgium; 3 Unit of Physiotherapy, Department of Health and Rehabilitation, Institute of Neuroscience and Physiology, Sahlgrenska Academy, University of Gothenburg, 405 30 Goteborg, Sweden; 4 Research Foundation—Flanders (FWO), 1000 Brussels, Belgium; 5 Rehabilitation Research Group, Department of Physiotherapy, Vrije Universiteit Brussel, 1090 Brussels, Belgium; 6 REVAL, Universiteit Hasselt, 3590 Diepenbeek, Belgium; 7 Interuniversity Centre for Health Economics Research (I-CHER), Department of Public Health (GEWE), Faculty of Medicine and Pharmacy, Vrije Universiteit Brussel, 1090 Brussels, Belgium; 8 Laboratory for Brain-Gut Axis Studies (LaBGAS), Translational Research in Gastrointestinal Disorders (TARGID), Department of Chronic Diseases and Metabolism (CHROMETA), Katholieke Universiteit Leuven, 3000 Leuven, Belgium; 9 Musculoskeletal Rehabilitation Research Group, Department of Rehabilitation Sciences, Faculty of Movement and Rehabilitation Sciences, Katholieke Universiteit Leuven, 3000 Leuven, Belgium; 10 Brain, Body and Cognition (BBCO), Faculty of Psychology and Educational Sciences, Vrije Universiteit Brussel, 1090 Brussels, Belgium; 11 Division of Rheumatology, University Hospitals Leuven, 3000 Leuven, Belgium; 12 Institute of Advanced Study, University of Warwick, Coventry CV4 7AL, UK; 13 Department of Psychology, University of Warwick, Coventry CV4 7AL, UK; 14 Department of Public Health and Primary Care, Centre for Environment and Health, Katholieke Universiteit Leuven, 3000 Leuven, Belgium; 15 Department of Movement and Sport Sciences, Faculty of Physical Education and Physiotherapy, Vrije Universiteit Brussel, 1090 Brussels, Belgium

**Keywords:** chronic pain, lifestyle, physical activity, stress management, nutrition, sleep management

## Abstract

Chronic pain is the most prevalent disease worldwide, leading to substantial disability and socioeconomic burden. Therefore, it can be regarded as a public health disease and major challenge to scientists, clinicians and affected individuals. Behavioral lifestyle factors, such as, physical (in)activity, stress, poor sleep and an unhealthy diet are increasingly recognized as perpetuating factors for chronic pain. Yet, current management options for patients with chronic pain often do not address lifestyle factors in a personalized multimodal fashion. This state-of-the-art clinical perspective aims to address this gap by discussing how clinicians can simultaneously incorporate various lifestyle factors into a personalized multimodal lifestyle intervention for individuals with chronic pain. To do so the available evidence on (multimodal) lifestyle interventions targeting physical (in)activity, stress, sleep and nutritional factors, specifically, was reviewed and synthetized from a clinical point of view. First, advise is provided on how to design a personalized multimodal lifestyle approach for a specific patient. Subsequently, best-evidence recommendations on how to integrate physical (in)activity, stress, sleep and nutritional factors as treatment targets into a personalized multimodal lifestyle approach are outlined. Evidence supporting such a personalized multimodal lifestyle approach is growing, but further studies are needed.

## 1. Introduction

Globally, chronic pain is the most prevalent disease, leading to significant disability and high socioeconomic burden [1,2]. Therefore, it is considered a public health disease and a major challenge to scientists, healthcare practitioners and the affected individuals. Breakthrough (neuroscience) research [3,4] led to the recognition by the World Health Organization of chronic pain as a disease characterized by functional and structural brain changes [3,4], neuroinflammation [5,6] and increased sensitivity of the central nervous system to sensory input (‘central sensitization’) [7,8].

In the context of any chronic condition, it is essential to investigate modifiable perpetuating factors in order to pinpoint potential treatment targets. Accumulating evidence indicates that lifestyle factors, including physical (in)activity, stress, inadequate sleep and an unhealthy diet, are linked to the severity of chronic pain through both direct mechanisms (such as neurophysiology) and indirect pathways (such as improvements in mood), and these associations persist across all age groups [9,10,11,12]. Furthermore, chronic pain is correlated with a reduced life expectancy [13,14], attributed in part to increased mortality from cancer and cardiovascular disease, as well as the misuse of opioid analgesics [14,15,16]. This connection is partially explained by unfavorable lifestyle factors like low physical activity levels, poor dietary habits and smoking [17,18]. Consequently, behavioral and lifestyle factors are increasingly acknowledged as perpetuating elements in the context of the severity of chronic pain and its consequences. As such, they are acknowledged treatment targets in chronic pain management. Yet, existing treatment approaches for individuals experiencing chronic pain frequently fail to fully address the diverse lifestyle factors potentially linked to chronic pain. They either neglect these factors altogether or address only them in a standardized manner, rather than offering a personalized and comprehensive multimodal lifestyle intervention [9,19]. To fully target these lifestyle factors, a paradigm shift from traditional interventions that focus solely on tissues and diseases to one that customizes the intervention to address these specific modifiable lifestyle factors is required. Such a paradigm shift, as supported by recent studies [12,20,21,22], holds the potential to enhance outcomes in pain severity and alleviate the psychological and socioeconomic impact associated with chronic pain.

The main aim of this state-of-the-art perspective is to elucidate how clinicians can comprehensively address diverse lifestyle factors concurrently within a personalized multimodal lifestyle intervention for individuals with chronic pain. To achieve full comprehensiveness, the initial section delineates the process of identifying relevant lifestyle factors linked to chronic pain during patient assessment—an essential step for designing personalized lifestyle management strategies. Given that addressing lifestyle factors typically necessitates behavioral changes, an approach to identifying determinants and barriers to behavioral lifestyle changes is also outlined. The second part of this perspective is dedicated to exploring the roles of exercise, physical (in)activity, sleep, psychological stress, nutrition and weight in chronic pain. It additionally delves into how these specific lifestyle factors can be addressed in a personalized manner within the context of pain management. It is important to mention that other lifestyle factors, like smoking, social (dis)connection, etc., will not be covered in detail in this paper.

## 2. Designing a Personalized Multimodal Lifestyle Intervention for Individuals with Chronic Pain

### 2.1. Identifying Relevant Lifestyle Factors

To personalize the lifestyle approach, clinicians need to identify relevant lifestyle factors for each patient that are associated with their chronic pain condition. This may encompass a set of broad inquiries related to various aspects of one’s lifestyle, such as asking questions like “Do you wake up feeling refreshed?” or “How frequently do you consume vegetables and fruits in your diet? What does your typical breakfast, lunch, or dinner consist of?” or “Do you experience frequent stress?” These questions are typically part of the standard initial assessment. If these initial questions reveal potential areas that require therapeutic attention, further probing and comprehensive assessments can be carried out. For more in-depth information, readers are encouraged to explore specialized publications dedicated to each of these specific lifestyle factors, which include specific guidance on assessing lifestyle factors, including the use of self-report measures [19,23,24,25,26,27].

Tailoring treatment approaches is essential for addressing relevant lifestyle factors effectively. For individuals dealing with chronic pain and coexisting insomnia, evidence-based treatments such as cognitive behavioral therapy for insomnia can be beneficial. Patients facing challenges in coping with daily stressors may find relief through participation in a stress management program. Moreover, those with chronic pain and comorbid overweight or obesity can benefit from incorporating a behavioral weight reduction program, involving changes in dietary and physical activity behaviors, into their rehabilitation regimen based on the current best evidence [23]. Also, patients with chronic pain and a normal weight who show poor dietary habits can benefit from dietary changes [28]. Regarding exercise and physical activity, the majority of chronic pain patients may require a physical activity and/or exercise intervention. However, like other lifestyle intervention programs, personalized approaches are essential for effective exercise and physical activity interventions.

### 2.2. Identifying Determinants and Barriers for a Behavioral Lifestyle Change

**Figure 1:**
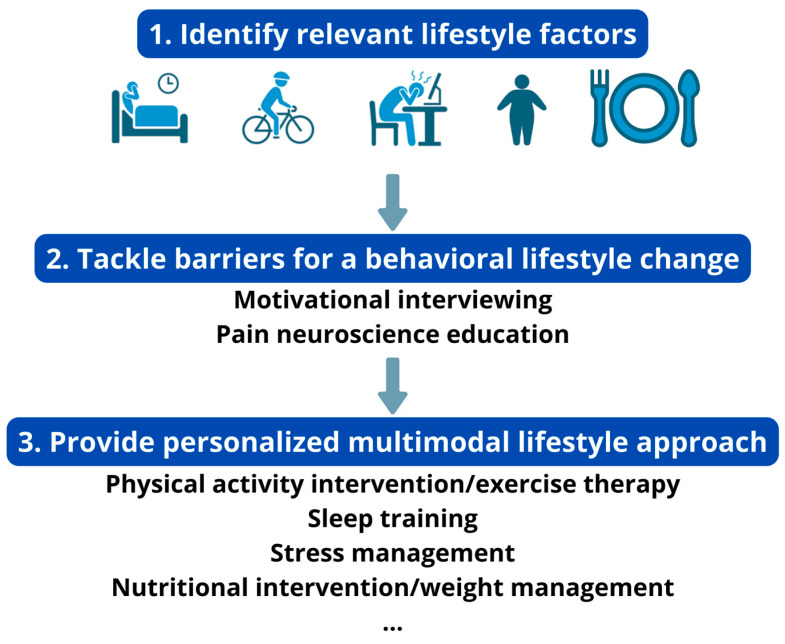
How to develop a personalized multimodal lifestyle intervention for chronic pain.

Engaging in a lifestyle approach implies a behavioral change (e.g., changing diet, becoming more physically active) from the patient (Figure 1). In general, individuals with chronic pain experience greater difficulty in engaging in positive health behaviors than those without pain [29]. Hence, clinicians need to identify possible barriers for engaging in the behavioral change that is required for adhering to a healthy lifestyle. For instance, predominant biomedical beliefs, fear of movement, heightened worry (in the context of pain), hypervigilance, (lack of) self-compassion, perceived injustice and poor acceptance are often seen in patients with chronic pain, and each of them can serve as a barrier for an adaptive lifestyle change [30,31,32,33,34]. Therefore, they should be identified and addressed before initiating the behavioral lifestyle intervention. For example, pain neuroscience education can change unhelpful pain beliefs into more adaptive ones, and thus address excessive worry and fear of movement [35]. When integrated in a cognitive behavioral program, it might further facilitate individuals with chronic pain to increase physical activity levels and engage in the exercise/physical activity lifestyle interventions [36].

While pain neuroscience education is not a comprehensive treatment in itself, it might prepare the patient for a behavioral lifestyle intervention [9]. In the context of this clinical perspective, clinicians are encouraged to incorporate insights into the pivotal role of lifestyle factors within their pain neuroscience education sessions with the patient, tailoring the approach based on the patient’s clinical profile and context. This involves educating the patient about the intricate connection between pain and quality of life and the impacts of physical (in)activity, stress tolerance, recuperative sleep and a healthy diet.

Additionally, integrating motivational interviewing into pain neuroscience education can further enhance behavioral change in individuals with chronic pain. Both conceptually and scientifically, motivational interviewing and pain neuroscience education emerge as complementary interventions that can aid individuals in adhering to treatment principles. While pain neuroscience education contributes to cognitive awareness, encompassing pain knowledge/beliefs and willingness, motivational interviewing primarily focuses on enhancing behavior awareness. Indeed, motivational interviewing seeks to create a perceived discrepancy between current behavior and personal goals, to navigate resistance and to foster self-efficacy [37]. The ultimate goal is to enhance the patient’s perceived competence and self-regulation, fostering intrinsic motivation for behavioral change [38]. In the context of a personalized multimodal lifestyle intervention, employing motivational interviewing implies that components of the physical activity/exercise, stress, sleep, or nutritional management are suggested by the patient through a guided question-and-answer process with the clinician. This way, it optimally serves the shared decision-making process [39]. Further guidance on integrating motivational interviewing with pain neuroscience education in chronic pain management, including the shared decision-making process and defining of patient-valued goals, is available in a detailed script elsewhere [39].

It is important to acknowledge that patients come with different backgrounds (e.g., health literacy, educational level, cultural background), making the shared decision-making process highly relevant to taking such differences into account. Additionally, it is plausible that some patients may not attain the intrinsic motivational level necessary to fully participate in all aspects of lifestyle management based on their clinical profile. Commencing lifestyle management components that necessitate behavioral changes without the patient’s consent and/or intrinsic motivation not only proves ineffective but also can undermine the therapeutic alliance [39,40].

Lastly, although the multimodal assessment of lifestyles might increase the effectiveness of care, the feasibility of a multimodal treatment plan should be considered during the shared decision-making process. It might be important to prioritize the lifestyle factors relevant to the treatment, to not overwhelm the patient and prevent non-participation in therapy or drop-out. The same holds for other conservative cares the patient might receive (e.g., psychology, osteopathy, physiotherapy, acupuncture, homeopathy and pharmacological interventions). In such cases, a ‘case manager’ (e.g., primary care practitioners or expert physical therapists) might be required to support the patient in balancing and prioritizing the various treatments, certainly if different treatments might induce conflicting information. For example, (partly) adhering to passive treatments might be a barrier to engage in active coping skills, which are necessary for behavioral change. Unless the various treatments are offered by an in-house interdisciplinary team, they increase the odds of receiving conflicting messages from various healthcare practitioners, reducing the chances of therapeutic success.

## 3. Physical Activity and Exercise Therapy within a Personalized Multimodal Lifestyle Intervention for Individuals with Chronic Pain

The paramount importance of achieving an appropriate level of physical activity for individuals with chronic pain is widely acknowledged. Extensive research on physical (in)activity in patients with chronic pain has revealed that the majority experience a reduction in overall physical activity levels due to the impact of chronic pain [41,42,43,44]. The more debilitating the pain, the lower the engagement in physical activity [45].

### 3.1. Patients Combine Avoidance and Persistence Behavior

Beyond the general decrease in physical activity among individuals with chronic pain, patients often adopt a combination of avoidance and persistence (or over-activity) behaviors [46,47], with specific avoidance or persistence behavioral patterns emerging based on personal beliefs, and motivational or contextual factors [25]. For example, a patient could be persisting in household chores, while simultaneously avoiding leisure-time physical activity.

Customizing the management of physical activity and exercise therapy to address specific activities that an individual finds challenging to manage is essential for patient-centered care [48]. This approach diverges from the common practice of applying one-size-fits-all physical activities and exercise programs in scientific studies, which exhibit limited effectiveness in improving pain severity, physical functioning and quality of life, and may even lead to adverse events [49]. Another factor contributing to the reported low effect sizes of one-size-fits-all exercise programs could be their lack of integration within a multimodal lifestyle program, coupled with educational and motivational interventions, as discussed earlier. Indeed, patients’ beliefs about pain, along with their perceived roles in life (e.g., work responsibilities) and life expectations (e.g., raising children), influence their attitudes and behaviors (e.g., avoiding sports activities or persisting at work and in the household) [50]. Addressing these pain-related cognitions, beliefs and behaviors within a context that is relevant to the patient is often a prerequisite for fostering behavioral changes in physical activity among individuals with chronic pain and their significant others.

### 3.2. Personalizing Physical Activity and Exercise Therapy Interventions

The avoidance of activities due to pain can be effectively tackled through behavioral graded exercise/activity or cognition-targeted exercise therapy. Behavioral graded exercises/activities are successful behavioral treatments that incorporate the principles of operant conditioning to elevate the patient’s daily physical activity and exercise levels [51]. It is a highly personalized approach, employing value-based goal setting for specific treatment objectives and individually tailoring the baseline and grading levels for performing valued daily activities or exercises [52]. Cognition-targeted exercise therapy includes introducing new exercises using motor imagery, integrating them with increasing complexity using a time-contingent progression and practicing them in different environments and contexts in order to maximize the transfer to daily situations [53]. ‘Cognition-targeted’ additionally implies addressing the patient’s cognitions about their problems during exercises, so that the patient obtains positive perceptions and expectations regarding the effects of the exercises on their pain and (most importantly) their ability to reach their self-defined long-term functional goals [53].

**Figure 2:**
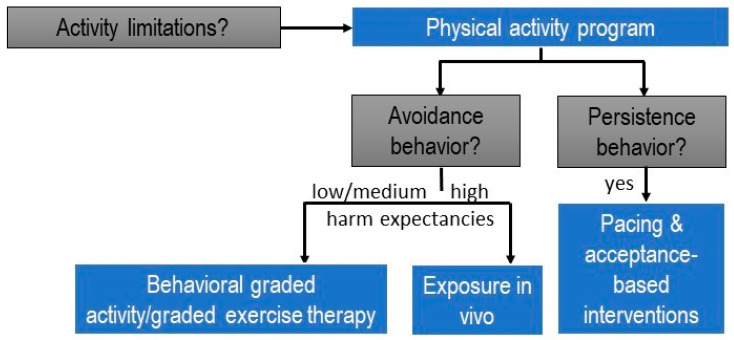
Clinical reasoning decision-making tree for designing physical activity programs within the personalized multimodal lifestyle interventions for individuals with chronic pain (modified from [54]).

Activities that are persisted require activity pacing self-management and acceptance-based interventions (Figure 2).

As an illustration, consider a patient with chronic low back pain who has ceased playing tennis but continues ironing, despite the activity causing significant stress and aggravating the pain. In such cases, it is advisable not to include ironing in a (graded) exercise/physical activity therapy program immediately. Instead, the patient should receive support through interventions such as stress management to address the stress and pain flares associated with ironing. Activity pacing self-management is also recommended, teaching the patient to distribute the activity across multiple shorter bouts and incorporate breaks to practice proposed relaxation skills [55]. To facilitate the patient’s return to playing tennis, we can approach the situation by asking open-ended questions like “What led to your decision to stop playing tennis?” Alternatively, we can employ a rating scale to assess the perceived fear of harm associated with playing tennis. For instance, we might phrase it as “On a scale from 0 to 100, could you rate the perceived harmfulness of playing tennis? Use 0 for not perceived as harmful at all, and 100 for an unbearable level of perceived harmfulness.” (Note: It is advisable to avoid using the word ‘fear’ to maintain a supportive therapeutic environment.) For tennis movements not reported as avoided due to a fear of harm or scoring below 70 (the cut-off value for a high level of perceived harmfulness [56]), and for activities performed without applied safety behaviors, effective approaches include behavioral graded activity/graded exercise and cognition-targeted exercise therapy [36]. For the specific movements performed while playing tennis that are perceived as harmful, scoring higher than 70/100 on a harmfulness scale or involving identified safety behaviors, it is appropriate to design specific behavioral experiments, incorporated into an exposure in vivo approach [57].

In summary, tailoring physical activity or exercise therapy for patients with chronic pain involves a meticulous selection of the suitable type of intervention. This customization should be applied not only to each patient but also to each specific activity limitation within relevant contexts, considering whether the patient employs avoidance or persistence strategies to manage pain.

## 4. Sleep Management within a Personalized Multimodal Lifestyle Intervention for Individuals with Chronic Pain

For many patients with chronic pain, sleep represents a crucial yet often overlooked lifestyle factor that offers considerable room for improvement [19,58,59]. Unfortunately, clinicians working with chronic pain patients tend to neglect the significance of sleep.

### 4.1. Insomnia Is Highly Prevalent in Individuals with Chronic Pain

In the absence of other intrinsic sleep disorders (e.g., apnea, restless leg syndrome) that should be ruled out and in the absence of inadequate opportunities or circumstances for sleep (e.g., shift work), insomnia in adults is defined as >30 min of sleep latency and/or minutes awake after sleep onset for >3 days/week for >3 months, accompanied by apparent daytime symptoms (e.g., memory problems, low mood) [60,61,62]. Insomnia is highly prevalent among individuals suffering from chronic pain, affecting 53% to 90% of adults [63,64,65,66] and approximately 50% of children [67] and adolescents [68,69] with chronic pain, leading to clinically significant degrees of insomnia.

The relationship between sleep disturbances and chronic pain is bidirectional [61,64]. Sleep disturbances not only act as a perpetuating factor for chronic pain [60] but also are associated with depressive symptoms, functional disability, increased healthcare utilization and diminished quality of life in adolescents with chronic pain [69,70,71]. A noteworthy observation is that after experiencing a better night of sleep, patients with chronic pain tend to spontaneously engage in more physical activity [72]. This highlights the potential effectiveness of interventions that incorporate sleep training for patients dealing with chronic pain and sleep-related issues.

### 4.2. Evidence-Based Treatment for Insomnia in Individuals with Chronic Pain

If left untreated, insomnia can pose a barrier to effective chronic pain management [61]. However, many contemporary pain treatment programs often offer little beyond the prescription of sedative pain/sleep medications to ‘address’ insomnia (e.g., gabapentin, an anti-epileptic drug, is used to enhance sleep; amitriptyline, a tricyclic anti-depressive drug, is prescribed to improve sleep) [65]. Nevertheless, the pharmacological management of insomnia is recommended only for short-term use and is associated with negative side effects [73]. Meta-analyses examining the effects of non-pharmacological treatments for insomnia, such as cognitive behavioral therapy for insomnia (CBT-i), in both cancer- and non-cancer-related chronic pain conditions have shown that sleep training yields significant and immediate improvements in sleep quality. These improvements are accompanied by relatively small enhancements in pain and fatigue, along with moderate reductions in depressive symptoms [74,75]. Importantly, the gains in sleep quality and fatigue are sustained at 1-year follow-ups [74]. For a more comprehensive understanding of insomnia and its (non-pharmacological) management in patients with chronic pain, additional information can be found elsewhere [19,58,76].

### 4.3. Stepped Care Approach for Targeting Insomnia in Patients with Chronic Pain

**Figure 3:**
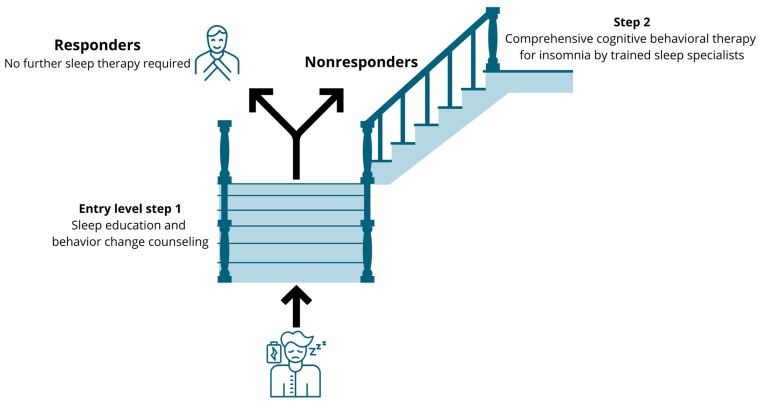
Suggestion for a stepped care approach to implement cognitive behavioral therapy for insomnia in patients with chronic pain and comorbid subthreshold insomnia.

Based on compelling effectiveness data, CBT-i is the gold standard treatment for insomnia [77]. It is a multicomponent treatment that challenges unhelpful sleep-related beliefs and behaviors and aims to replace them with appropriate beliefs and behaviors. Yet, CBT-i remains underused in clinical settings due to several implementation barriers [78]. Accessibility to CBT-i is extremely limited due to several barriers [79], including a shortage of CBT-i specialists and lack of physicians trained in sleep problems [80,81]. This creates opportunities for other (allied) healthcare practitioners such as occupational therapists, physiotherapists and nurses to fill this implementation gap, at the very least in a stepped care model, with each step consisting of evidence-based sleep management components [82]. The stepped care model implies that an ’entry level‘ treatment should be readily accessible, be delivered at the lowest level of therapeutic intensity, inconvenience patients the least, be provided at the lowest cost and require the least amount of specialist time [83]. Sleep education and behavior change counseling (including, sleep hygiene recommendations) meet all of these criteria [84], and comprise the replacement of sleep-interfering behaviors with sleep-promoting behaviors through sleep education and behavior change counselling [85]. For instance, it includes explaining that clock monitoring in bed, daytime sleeping, food and beverage consumption and intense exercise before bedtime may disrupt sleep initiation and maintenance [60,61,85,86]. Sleep hygiene recommendations also include explaining how patients can improve their sleep environment such as bedroom darkness, temperature, noise/quietness and humidity [60,61,85,86]. A stepped care approach that delivers components of CBT-i to patients with chronic pain can be suggested (Figure 3). This approach implies that those who do not benefit (enough) from the first ‘entry level’ treatment will obtain access to comprehensive CBT-i provided by a trained sleep specialist (step 2). Indeed, sleep education and mere behavior change counseling are significantly less efficacious than CBT-i for treating sleep problems in patients with a clinical level of insomnia [87]. Therefore, in patients with clear clinical insomnia, it might be preferred to directly offer the full CBT-i program to not delay high-quality care. As such, the stepped approach might be valuable for people with subthreshold levels of insomnia (e.g., no daytime symptoms of disturbed nighttime sleep) or with minor sleep problems.

### 4.4. Sleep Disturbances as a Physiological Stressor Requiring Stress Management

Insomnia and sleep disturbances constitute important examples of physiological stressors [88]. In patients with chronic pain [65], stress and sleep are consistently related, as supported by findings from numerous chronic pain studies reporting strong associations between anxiety levels and insomnia severity [89,90]. Daily life stress such as worries about the next morning’s workload can negatively impact sleep [91]. Increased nighttime arousal and decreased sleep efficiency are among the most sensitive sleep variables in response to stressors [91]. Therefore, the next section explains the role of stress as an additional cardinal lifestyle factor to be considered in personalized multimodal lifestyle interventions for patients with chronic pain.

## 5. Stress Management within a Personalized Multimodal Lifestyle Intervention for Individuals with Chronic Pain

### 5.1. Stress Intolerance Linked to Dysfunctional Physiological Stress Response Systems in Patients with Chronic Pain

Stress is defined as the effort of living organisms to maintain an internal dynamic balance, commonly known as homeostasis [92]. Any element, being either physical, psychosocial or emotional that poses a challenge to homeostasis can be categorized as a stressor [92]. Severe persisting stress [93,94,95] stands as a well-established lifestyle factor crucial in sustaining chronic pain [9]. The perception of stress is highly subjective, with individuals exhibiting varied responses to the same stressors [24]. Consequently, the stress response is contingent upon both the perceived stress and the characteristics of the stressor, including its nature, duration and intensity [96,97,98]. In individuals with chronic pain, stress is commonly linked to the deterioration of pain symptoms and the development of stress-induced hyperalgesia [24]. Much like insomnia, the impact of stress on individuals living with chronic pain extends beyond nociceptive modulation, influencing additional symptoms such as fatigue and cognitive disturbances [99,100]. As a result, the term “stress intolerance” has been introduced to describe the aggravation or onset of symptoms in response to stress [24]. Individuals enduring chronic pain often exhibit dysfunctional physiological stress response systems [101,102,103,104,105,106,107], encompassing both the short- (such as the sympathetic nervous system) and long-term (such as the hypothalamus–pituitary–adrenal axis) stress response systems (reviewed in [24]). Likewise, brain regions known to support allostasis [108] are frequently found to exhibit altered functional behavior across various chronic pain populations [109,110,111,112,113]. In a clinical context, this presents as a reduced capacity to cope with stress in everyday experiences [114].

### 5.2. Improving Stress Tolerance in Patients with Chronic Pain

Stress management should be considered in a personalized multimodal lifestyle intervention for patients with chronic pain and difficulties coping with everyday stressors, as supported by available evidence [115,116,117]. Such stress management typically starts by educating the patient about the role of stress (in relation to pain), which can fit nicely into pain neuroscience education as explained above [118]. Patient education and reassurance can reduce their distress and change their attitudes toward pain [119]. If one can adjust their perceptions regarding the role of stress and recognize how the suggested approaches can aid in enhancing their stress response system, there may be an increased likelihood of better adherence to stress self-management. It is crucial to acknowledge in stress management that inherent stress response systems may not operate optimally, necessitating external support for more effective coping with everyday stressors [9]. Cognitive behavioral therapy targeting pain interference, stress and disability can also be employed, in the attempt of reducing factors contributing to the pain experience [120,121,122]. In addition to stress education, stress management typically entails a variety of individually tailored stress coping strategies, varying from relaxation skills (e.g., Jacobson’s progressive muscle relaxation, mindfulness, visualization and breathing exercises), cognitive approaches to cope better with stress, the identification of relevant stressors and ‘uplifts’, etc. Detailed manuals on how to provide stress management to patients with chronic pain are provided elsewhere [118].

Ultimately, stress management should not be perceived as a completely isolated entity or element; instead, it should be viewed as a continuous thread woven throughout the entire multimodal lifestyle intervention [118]. In line with this, it should be considered that an unhealthy diet can serve as a stressor too, which, in turn, can decrease stress tolerance.

## 6. Nutritional Interventions and Weight Management within a Personalized Multimodal Lifestyle Intervention for Individuals with Chronic Pain

Diet represents another modifiable lifestyle factor of significant importance to patients with chronic pain [38]. Poor dietary habits can be considered as adverse lifestyle factors that partly account for the observed excess mortality among people with chronic pain [17,18]. Growing evidence suggests that nutrition should be considered within a personalized approach to pain management [20]. Within this view, it is important to emphasize that lifestyle factors such as diet and physical activity often fluctuate in time [123,124,125].

### 6.1. Chronic Pain: Part of the Obesity Pandemic?

Poor dietary habits often—but not always—relate to overweight or obesity [126]. The widespread prevalence of overweight and obesity poses a global health challenge, acknowledged as a primary public health issue in developed nations [127,128]. Meta-analyses affirm a positive association between overweight, obesity and low back pain [126,128,129,130,131,132,133], with a higher BMI and fat mass correlating with increased prevalence of chronic pain [134]. Furthermore, overweight and obesity contribute to more intense and debilitating chronic pain, as evidenced by dose–response relationships between pain intensity, disability, BMI, waist circumference, percent fat and fat mass in individuals with chronic low back pain [132]. In terms of socioeconomic impact, overweight and obesity correlate not only with persistent chronic pain but also with elevated healthcare-seeking rates for pain-related issues [126]. Individuals who are obese or overweight and experiencing chronic pain are likely to have more intricate health needs that necessitate a focus on behavioral lifestyle factors [135].

### 6.2. Adding Nutritional Interventions or Weight Management to Pain Management

Collectively, unhealthy dietary patterns, overweight and obesity are increasingly acknowledged as viable targets for therapeutic intervention in individuals with chronic pain [131,132,135,136,137]. However, few contemporary treatment programs address diet or weight, and the management of the latter can be complex in these individuals [138]. This is a notable limitation, considering a recent meta-analysis validating that nutritional interventions, particularly modifications to dietary patterns and specific nutrients, lead to substantial pain relief in individuals with chronic pain [139]. Studies involving overweight and obese adults with knee osteoarthritis demonstrate that a combination of diet and exercise therapy can yield moderate pain relief and improved physical function [140]. Notably, in a more recent randomized controlled trial (2020), three interventions were compared: exercise alone, intensive diet-induced weight loss alone and a combination of intensive diet-induced weight loss with exercise [141]. The study revealed that in comparison to exercise alone, diet-induced weight loss led to substantial reductions in load bearing at the hip, knee and ankle joints. However, when diet was combined with exercise, these reductions were less pronounced, although they remained significantly superior to the results achieved with exercise alone. Additionally, it is worth noting that diet and exercise therapy, when integrated into standard care, have proven to be cost-effective for such patients [142].

Weight management programs should include changes in dietary and physical activity behaviors (i.e., a self-management behavioral approach to balance caloric intake and physical activity) to comply with evidence-based standards rather than strict and harsh calorie restriction diets [137]. This should involve the integration of behavioral theories and constructs to support and facilitate energy balance-related behavior [143]. As emphasized in the American College of Sports Medicine (ACSM) Position Stands, the combination of a moderate dietary restriction and a physical activity program is effective and yields enduring outcomes [144]. Adopting a self-management approach to enhance behaviors related to energy balance can be seamlessly incorporated into the previously outlined cognition-targeted exercise therapy program. This implies that assessing daily physical activity and exercise levels will be geared toward not only weight reduction but also the simultaneous improvement of pain-related cognitions. Consequently, the exercises and daily physical activities can be aligned with the principles of cognition-targeted treatment as well as the personalized approaches for weight reduction. More detailed recommendations to support clinicians in providing personalized nutritional interventions in patients with chronic pain are provided elsewhere [20].

It is crucial to emphasize that individuals experiencing chronic pain, even those maintaining a healthy weight, may find value in nutritional interventions. Research indicates that the dietary quality of individuals with chronic pain tends to be lower than that of those without pain [139,145,146]. To address this, assessing and monitoring the patient’s dietary habits through a food diary can offer various avenues to enhance diet quality. A personalized approach is essential in this regard.

While specific recommendations may vary based on individual needs, general guidelines for nutritional interventions in chronic pain advocate for the inclusion of colorful fruits and vegetables, along with an adequate intake of high-quality fats to mitigate inflammation and oxidative stress [139]. Additionally, preventing deficiencies in Vitamin D, Vitamin B12 and Magnesium has the potential to contribute to pain alleviation [139,147,148]. Lastly, ensuring sufficient fiber intake proves significant for promoting proper digestion, maintaining a healthy microbiome, managing weight and subsequently influencing chronic pain [139].

## 7. Other Lifestyle Factors to Consider within Personalized Multimodal Chronic Pain Management

In addition to physical (in)activity, stress, sleep and diet, smoking represents an important lifestyle factor for some patients with chronic pain. For instance, an observational study found that individuals tend to smoke more cigarettes when experiencing higher pain levels and make fewer attempts to quit smoking during such periods [149]. This behavior can be attributed to the acute pain-relieving effect of nicotine, which makes it challenging for them to quit due to the pleasurable sensations it provides [150]. Despite its short-term analgesic benefits, tobacco smoking is associated with chronic pain intensity and prevalence in the long run [134,150,151,152]. This highlights the importance of including pain neuroscience education and motivational interviewing in comprehensive smoking cessation strategies to improve the individual’s understanding of the relationship between pain and smoking, potentially increasing their commitment to a smoking cessation program [149].

Smoking status in individuals with chronic pain is associated with alcohol-drug and opioid dependence [153,154]. When smoking and alcohol consumption are combined, their negative effects are compounded due to the alcohol’s ability to impede the body’s capacity to metabolize the carcinogenic compounds found in cigarettes [155]. Furthermore, it is worth noting that alcohol initially has an acute analgesic effect [156,157], which can elevate the risk of alcohol abuse in individuals with chronic pain [156,158].

Additionally, it is essential to consider the significant role of psychosocial factors in individuals abusing alcohol, which is often linked to abnormalities in the brain’s reward system [159]. Recent studies have shown that chronic pain patients with high levels of pain catastrophizing are more likely to engage in heavy drinking [160]. This underscores the importance of integrating pain neuroscience education with information about these lifestyle factors, as it may enhance the patient’s understanding about pain while helping to reduce maladaptive thought patterns that may perpetuate unhealthy behaviors.

However, evidence supporting the use of interventions targeting smoke cessation or alcohol abuse in patients with chronic pain is scarce.

One last lifestyle factor that was not yet highlighted in this manuscript is social (dis)connection, which refers to the state of interpersonal relationships and interactions within a community or society. Social connection typically implies positive and meaningful relationships with others, which fosters a sense of belonging and support. On the other hand, social disconnection suggests a lack of these relationships, which leads to feelings of isolation, loneliness and detachment from others. Evidence shows that low social connectedness is linked to higher pain reporting, and that an association exists between the level of social support and emotional distress [161,162]. Consequently, clinicians must not overlook the crucial role of the social dimension when considering the biopsychosocial framework in the management of (chronic) pain.

## 8. Conclusions

To integrate the available evidence on lifestyle factors and chronic pain into clinical practice, clinicians are advised to screen for unhelpful beliefs and behaviors as key determinants of an unhealthy lifestyle, and address them accordingly; for instance, by providing pain neuroscience education with integrated motivational interviewing. This prepares and motivates patients to engage in a personalized multimodal lifestyle approach, potentially comprising of physical activity/exercise therapy in combination with sleep training, stress management, nutritional interventions and weight management (depending on the patient’s current lifestyle, preferences and characteristics). Evidence supporting such a paradigm shift from a tissue- and disease-based approach toward a personalized multimodal lifestyle intervention for chronic pain is growing [12,20,21], but further studies are needed. Such research efforts hold the potential to improve outcomes and decrease the psychological and socioeconomic burden of chronic pain globally.

## Data Availability

No new data were created or analyzed in this study. Data sharing is not applicable to this article.
